# Supplementary material for: Alkali-Treated Titanium Coated with a Polyurethane, Magnesium and Hydroxyapatite Composite for Bone Tissue Engineering
Source: Nanomaterials (Basel). 2021 Apr 27;11(5):1129. doi: 10.3390/nano11051129 (PMC8145718; doi:10.3390/nano11051129)
Supplement: Supplementary file 1 [file nanomaterials-11-01129-s001.zip › nanomaterials-1163231-supplementary-for conversion.pdf]

Supporting information

For

Alkali-Treated Titanium Coated with a Polyurethane, Magnesium and Hydroxyapatite Composite for Bone Tissue Engineering

Mahmoud Agour [1,†], Abdalla Abdal-hay [2,3,*,†], Mohamed K. Hassan [1,4], Michal Bartnikowski [2] and Sašo Ivanovski [2,*]

[1] Department of Production Engineering and Design, Faculty of Engineering, Minia University, Minia 61112, Egypt; agour.mahmoud@gmail.com (M.A.); mkibrahiem@uqu.edu.sa (M.K.H.)

[2] Centre for Orofacial Regeneration, Reconstruction and Rehabilitation (COR3), School of Dentistry, Herston Campus, The University of Queensland, 288 Herston Road, Herston, QLD 4006, Australia; michal.bartnikowski@uq.edu.au (M.B.)

[3] Department of Engineering Materials and Mechanical Design, Faculty of Engineering, South Valley University, Qena 83523, Egypt

[4] Department of Mechanical Engineering, College of Engineering, Umm Al-Qura University (UQU), Mecca 24381, Saudi Arabia

* Correspondence: abdalla.ali@uq.edu.au (A.A.-h.); s.ivanovski@uq.edu.au (S.I.)

† Those authors contributed equally to this work

Keywords: Magnesium and its alloys; Hydroxyapatite; Surface modifications; Titanium implants, corrosion analysis; Biocompatibility; Bioactivity

2.1. Sample Preparation

Commercial pure Titanium sheet with Grade 1 which was purchased from William Gregor Ltd, London, UK was cut in a square shape with dimensions of 12 × 12 × 2 mm^3. The samples were then wet grinded in series with 180 to 1200 grit silicon carbide (SiC) waterproof paper, and then were polished to a mirror finish with a 1.0 µm monocrystalline diamond suspension. Subsequently, the samples were cleaned ultrasonically in ethanol and distilled water for 10 minutes at volume ratio 1:1. After the polishing and washing processes, the samples were dried at room temperature [1–6].

2.2. Wet Chemical Synthesis of HAp

A HAp nanopowder was prepared using a wet chemical procedure, as described previously [7]. A 0.6 M $(NH_4)_2HPO_4$ solution was addiction average of 0.4 mL·min^{-1} to a 1 M solution of $Ca(NO_3)_2$ $4H_2O$ under intense stirring (The Ca/P ratio was 1.67 when these solutions were mixed, to supply stoichiometric HAp); an ammonium solution was used to adjust the pH of the final solution to pH 11. After intense stirring at room temperature (RT) for 3 h, the milky solution was heated to 90 °C for 1.5 h under continuous stirring. The resultant precipitate was maintained under vigorous stirring for twenty-four hours at RT to form a homogeneous solution. Finally, the resulting milky solution was filtered, and the precipitate was washed several times with a mixture of water and ethanol (volume ratio = 1:1) until the pH was neutralized (i.e. ~7). The final precipitated HAp nanopowder was dispersed in ethanol to reduce agglomeration. The synthesized powder was then dried under vacuum for 24 h, then calcined at 650 °C for 4 h in ambient air, with a heating rate of 20 ºC min^{-1}.

2.3. Characterization

The morphology of the surfaces was examined using a scanning electron microscope (SEM, JSM 5900, JEOL, Tokyo, Japan) with an acceleration voltage of 10 kV, equipped with an energy dispersive spectroscope (EDS). All the polymer coatings were sputter coated with a 10 nm layer of gold prior to examination, to enhance the surface conductivity and thus image quality. For measuring the coating thickness of the samples, samples were embedded in epoxy resin and ground and polished, prior to SEM imaging of cross-sections.

The surface composition of the samples was examined using XRD, (X′pert Pro-mrd PANalytical Co., Eindhoven, The Netherlands) with Cu Kα (k = 1.54056 Å) radiation over a 2 h range of angles from 10 to 70°. Fourier transform infrared FTIR, (Renishaw, Dongguan, China) spectroscopy in transmission mode was used to investigate the compounds formed on the substrate surface. The signal resolution of the FTIR was 1.0 cm^{-1} and a minimum of 16 scans was obtained and averaged within the range of 400–4000 cm^{-1}. The films was investigated using simultaneous thermogravimetric differential scanning calorimetry (TGA/DSC, New Castle, DE, USA) for thermal behavior, which measured both heat flow and weight changes of the samples as a function of temperature.

2.5. Adhesion performance

The adhesion strength of the coating was measured using a crosscut test with a hot crosshatch Adhesion Tester (QFH-HG600 hot Cross hatch adhesion tester instruction cross-cut tester Kit, NBC practical, New Castle, DE,USA). This instrument is designed and manufactured according to the standard ISO 2409-1992, and applicable to GB/T9286-98, BS3900E6/ASTM D3359, where the cutting tool is fitted with a blade containing eight teeth spaced 1.0 mm apart. Coated samples were secured to the lab bench by a clamp and the cutting tool was used to make a cross-cut pattern through the coating at 90° angles. The coating was lightly brushed with a soft brush after each cut to remove any excess debris from the surface. Transparent tape was applied to the cut surface, rubbed on with a hard object to ensure a tight adhesion, and then removed after 90 s, to attempt to induce coating delamination. Samples were evaluated under a lighted magnifying glass (7 ×) and rated according to the ASTM rating scheme.

2.6. Osteoblast cell response

Mouse osteoblast-like cells (MC3T3-E1) were obtained from the American Type Culture Collection (Manassas, VA, USA). The culture media was prepared by adding 10% fetal bovine serum (FBS, Gibco Co., San Diego, CA, USA), 500 $mg \cdot mL^{-1}$ penicillin (Gibco Co., San Diego, CA, USA), and 500 $U \cdot mL^{-1}$ streptomycin (Gibco Co., San Diego, CA, USA) to α-minimal essential media (MEM; Gibco, Carlsbad, CA, USA). Cells were cultured in T-75 flasks with MEM, at 37°C in a humidified atmosphere of 5% CO_2-95% air. A cytotoxicity test was conducted using a Cell Counting Kit-8 (Enzo Life Sciences Inc., Farmingdale, NY, USA)

according to the manufacturer's instructions, on day 1 and 3 of the culture. Briefly, the culture media was replaced with the WST-8 cell proliferation reagent [2-(2-methoxy-4-nitrophenyl)-3-(4-nitrophenyl)-5-(2,4-disulfophenyl)-2H-tetrazolium, monosodium salt] for 1.5 h at 37°C.

Cell morphology was examined using a scanning electron microscope (SEM, JSM 5900, JEOL, Tokyo, Japan). The metabolic activity (cells proliferation) of treated and untreated Ti samples were assessed by MTT assay kit as described in our previous publications [10–12].

The differentiation of osteoblast cells was evaluated through the expression of alkaline phosphatase (ALP) activity using the Sensolyte® pNPP ALP Assay Kit (AnaSpec, Fremont, CA, USA) after 8- and 14-days of culture. Firstly, the cells that were cultured on the samples were washed with 1 × buffer (AnaSpec, Fremont, CA, USA). To each sample, 200 μL of 0.2% Triton X-100 (AnaSpec, Fremont, CA, USA) was then added to lyse the cells. The lysate was collected in a micro centrifuge tube using a cell scraper, incubated at 4 °C for 10 min under agitation and centrifuged at 2500 rpm for 10 min at 4 °C. A 50 μL aliquot of supernatant was mixed with 50 μL of p-nitrophenyl phosphate (pNPP) (AnaSpec, Fremont, CA, USA). The mixture was incubated at 37 °C for 30 min and the reaction was stopped by adding 50 μL of NaOH solution (AnaSpec, Fremont, CA, USA) in each well. The final solution showed a yellow-colored product due to conversion of p-nitrophenyl phosphatase (pNPP) to p-nitrophenol (pNP) and was measured spectrophotometrically at 405 nm (microplate reader, Molecular Devices, SpectraMax model, San Jose, CA, USA). The ALP activity was normalized to a standard curve obtained with known ALP concentrations of 0-200 ng·mL^{-1}. This assay was completed in triplicate.

Mg^{2+} ion release from the composite film was investigated by placing the sample in sterile micro-centrifuge tubes (15 mL) with 6 mL α-MEM culture medium, and incubated at 37 °C in a humidified atmosphere of 95% air and 5% CO_2 for 3, 5, 8 and 14 d. At the end of each incubation period, the solution from each sample was collected. The release rates of ions were determined by Inductively coupled plasma atomic emission spectroscopy (ICP-AES, (Waltham, Massachusetts, USA) at back pressure of 216 kPa, and a 0.70

$L \cdot min^{-1}$ flow rate. Statistical significance was analyzed for all groups using a one-way ANOVA test, except the negative group. Statistical significance was considered present when the p value was less than 0.05.

References

1. Song, G.; Song, S., A possible biodegradable magnesium implant material. *Adv. Eng. Mater.* **2007**, *9*, 298–302.
2. Kokubo, T.; Kim, H.-M.; Kawashita, M., Novel bioactive materials with different mechanical properties. *Biomaterials* **2003**, *24*, 2161–2175.
3. Lu, X.; Wang, Y.-b.; Liu, Y.-r.; Wang, J.-x.; Qu, S.-x.; Feng, B.; Weng, J., Preparation of HA/chitosan composite coatings on alkali treated titanium surfaces through sol–gel techniques. *Mater. Lett.* **2007**, *61*, 3970-3973.
4. Cui, W.; Jin, L.; Zhou, L., Surface characteristics and electrochemical corrosion behavior of a pre-anodized microarc oxidation coating on titanium alloy. *Mater. Sci. Eng., C* **2013**, *33*, 3775–3779.
5. Xie, Y.; Ao, H.; Xin, S.; Zheng, X.; Ding, C., Enhanced cellular responses to titanium coating with hierarchical hybrid structure. *Mater. Sci. Eng., C* **2014**, *38*, 272–277.
6. Abdal-Hay, A.; Hamdy, A.S.; Khalil, K.A.; Lim, J.H., A novel simple one-step air jet spinning approach for deposition of poly (vinyl acetate)/hydroxyapatite composite nanofibers on Ti implants. *Mater. Sci. Eng., C* **2015**, *49*, 681–690.
7. Abdal-hay, A.; Agour, M.; Kim, Y.-K.; Lee, M.-H.; Hassan, M.K.; El-Ainin, H.A.; Hamdy, A.S.; Ivanovski, S., Magnesium-particle/polyurethane composite layer coating on titanium surfaces for orthopedic applications. *Eur. Polym. J.* **2019**, *112*, 555–568.
8. Abdal-hay, A.; Barakat, N.A.; Lim, J.K., Hydroxyapatite-doped poly (lactic acid) porous film coating for enhanced bioactivity and corrosion behavior of AZ31 Mg alloy for orthopedic applications. *Ceram. Int.* **2013,** *39*, 183-195.
9. Nayar, S.; Sinha, M.; Basu, D.; Sinha, A., Synthesis and sintering of biomimetic hydroxyapatite nanoparticles for biomedical applications. *J. Mater. Sci.-Mater. Med.* **2006**, *17*, 1063–1068.
10. Abdal-hay, A.; Agour, M.; Kim, Y.-K.; Lee, M.-H.; Hassan, M.K.; El-Ainin, H.A.; Hamdy, A.S.; Ivanovski, S., Magnesium-particle/polyurethane composite layer coating on titanium surfaces for orthopedic applications. *Eur. Polym. J.* **2018**, *112*, 555–568.
11. Abdal-hay, A.; Dewidar, M.; Lim, J.K., Biocorrosion behavior and cell viability of adhesive polymer coated magnesium based alloys for medical implants. *Appl. Surf. Sci.* **2012**, *261*, 536–546.
12. Abdal-hay, A.; Hwang, M.-G.; Lim, J., In vitro bioactivity of titanium implants coated with bicomponent hybrid biodegradable polymers. *J. Sol.-Gel Sci. Technol.* **2012**, *64*, 756-764.
